# A new type of retropancreatic fascia hernia in the supramesocolic space preoperatively misdiagnosed as a diaphragmatic hernia: report of two cases

**DOI:** 10.1186/s40792-023-01586-y

**Published:** 2023-01-11

**Authors:** Yoichi Nakagawa, Hiroo Uchida, Satoshi Makita, Kazuki Yokota, Akinari Hinoki, Chiyoe Shirota, Takahisa Tainaka, Wataru Sumida, Hizuru Amano, Seiya Ogata, Aitaro Takimoto, Shunya Takada, Takuya Maeda, Yousuke Gohda

**Affiliations:** 1 Department of Pediatric Surgery, Nagoya University Graduate School of Medicine, 65 Tsurumai-Cho, Showa-Ku, Nagoya, Aichi 466-8550 Japan; 2 Department of Gastrointestinal and Pediatric Surgery, Mie University Graduate School of Medicine and Faculty of Medicine, 2-174 Edobashi, Tsu, Mie 514-8507 Japan; 3 Department of Rare/Intractable Cancer Analysis Research, Nagoya University Graduate School of Medicine, 65 Tsurumai-Cho, Showa-Ku, Nagoya, 466-8550 Japan

**Keywords:** Retropancreatic fascia hernia, Retropancreatic fascia, Diaphragmatic hernia, Supramesocolic space, Retroperitoneal hernia

## Abstract

**Background:**

We encountered two cases of a new type of retroperitoneal hernia. We herein report the unique features of these cases.

**Case presentation:**

Case 1: A Japanese girl was born at a gestational age of 37 weeks, weighing 2550 g. She underwent laparotomic left diaphragmatic hernia repair for a left Bochdalek hernia at the age of one day. The postoperative course was uneventful; however, chest radiography at the age of 35 days revealed bowel gas in the mediastinum, while computed tomography exhibited intestinal prolapses from the medial side of the mesh into the thoracic cavity. Reoperation was performed at the age of 77 days, showing that the defect hole was not at the diaphragm but in the absence of retropancreatic fascia, which was connected to the posterior mediastinum from the supramesocolic space. The mediastinum space was closed with a suturing spine and artificial mesh, and the defect hole in the pancreatic body was sutured. Case 2: A Japanese boy was born at a gestational age of 40 weeks, weighing 3502 g. He was diagnosed with a left diaphragmatic hernia at birth and underwent laparotomy at the age of two days. Operative findings showed no defect hole in the diaphragm, and no intestine was observed in the abdominal cavity. After close observation of the abdominal cavity, the intestine was found around the pancreatic body, and manual reduction of the intestine was performed. The defect hole existed in the absence of the retropancreatic fascia, which was connected to the extra-pleural space. The defect hole in the pancreatic body was sutured and closed with a non-absorbable thread.

**Conclusions:**

We assumed that our cases were a new type of retroperitoneal hernia, which we named “retropancreatic fascia hernia”.

## Background

A congenital diaphragmatic hernia occurs 2.3 in 10,000 births [1]. It presents with respiratory discomfort and pulmonary hypertension; however, it is asymptomatic in some cases. We encountered two cases of congenital retroperitoneal hernias that presented with congenital diaphragmatic hernias. Considering the location of the hernia orifices in our patients, we assumed that these are new types of retroperitoneal hernia and named it “retropancreatic fascia hernia”. Herein, we report two cases of retropancreatic fascia hernia preoperatively diagnosed as a congenital diaphragmatic hernia.

## Case presentation

### Case 1

**Fig. 1:**
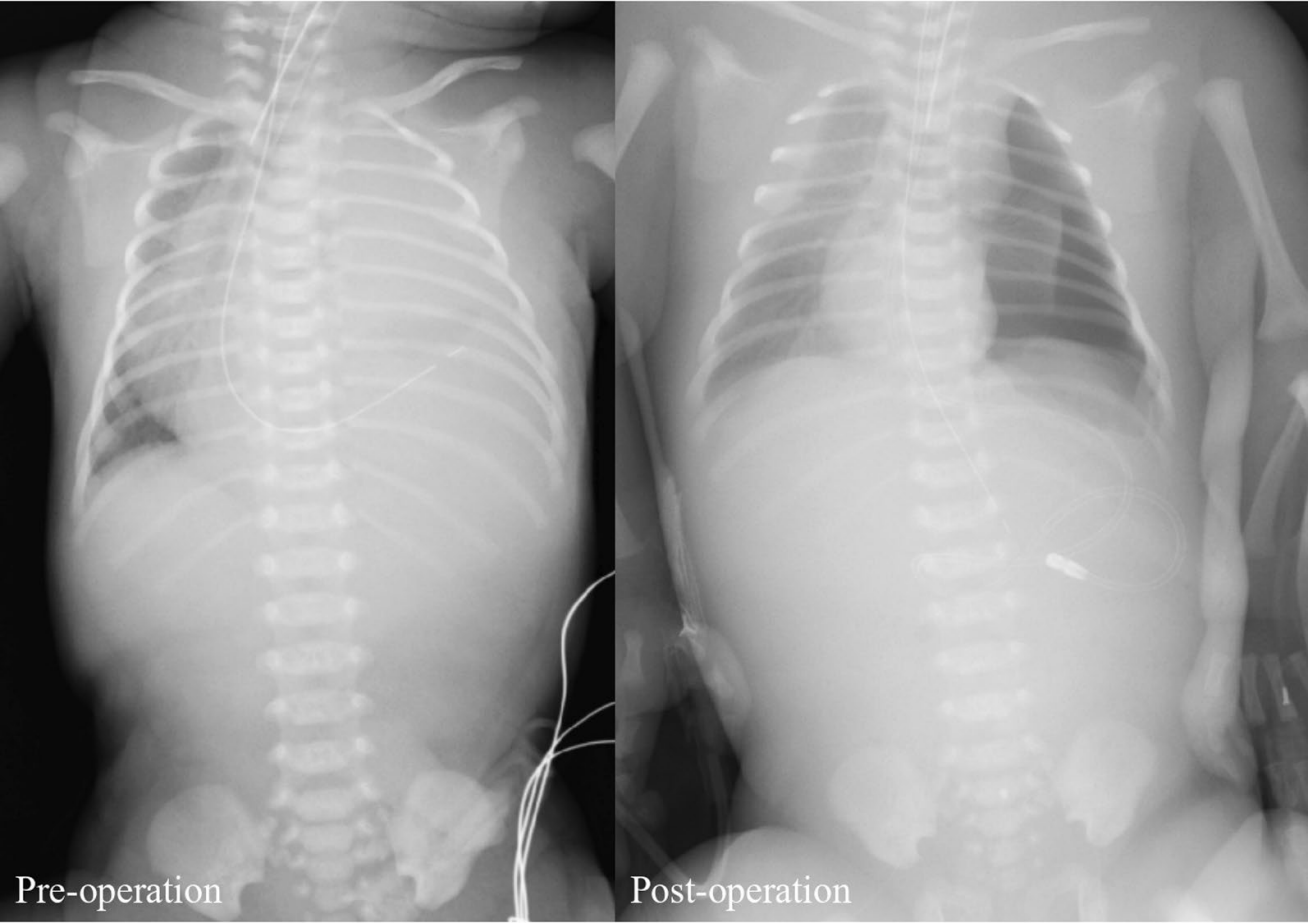
Chest radiograph showing a left diaphragmatic hernia. Preoperative chest radiograph reveals left diaphragmatic hernia. Postoperative chest radiograph reveals that the prolapsed organs are reduced

**Fig. 2:**
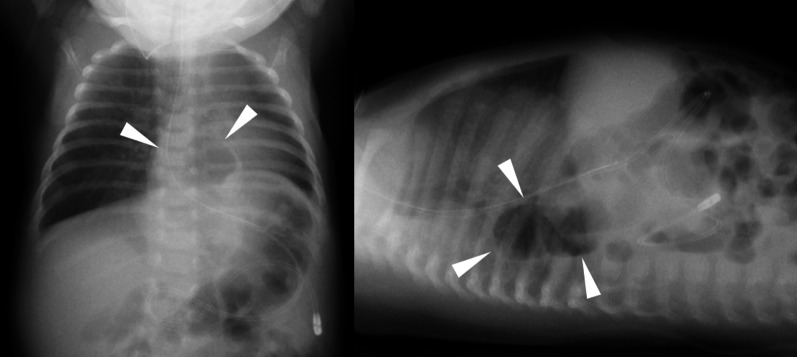
Chest radiograph of a suspected recurrent left diaphragmatic hernia. Chest radiograph on postoperative day 35 shows bowel gas is present in the thoracic cavity (arrowhead)

**Fig. 3:**
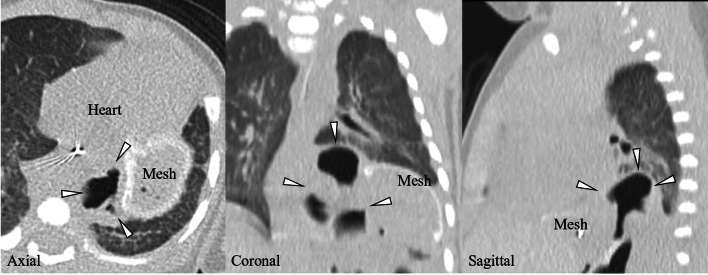
Computed tomography in case 1. Computed tomography shows intestine or colon is prolapsed into the thoracic cavity at the medial side of artificial mesh (arrowhead)

**Fig. 4:**
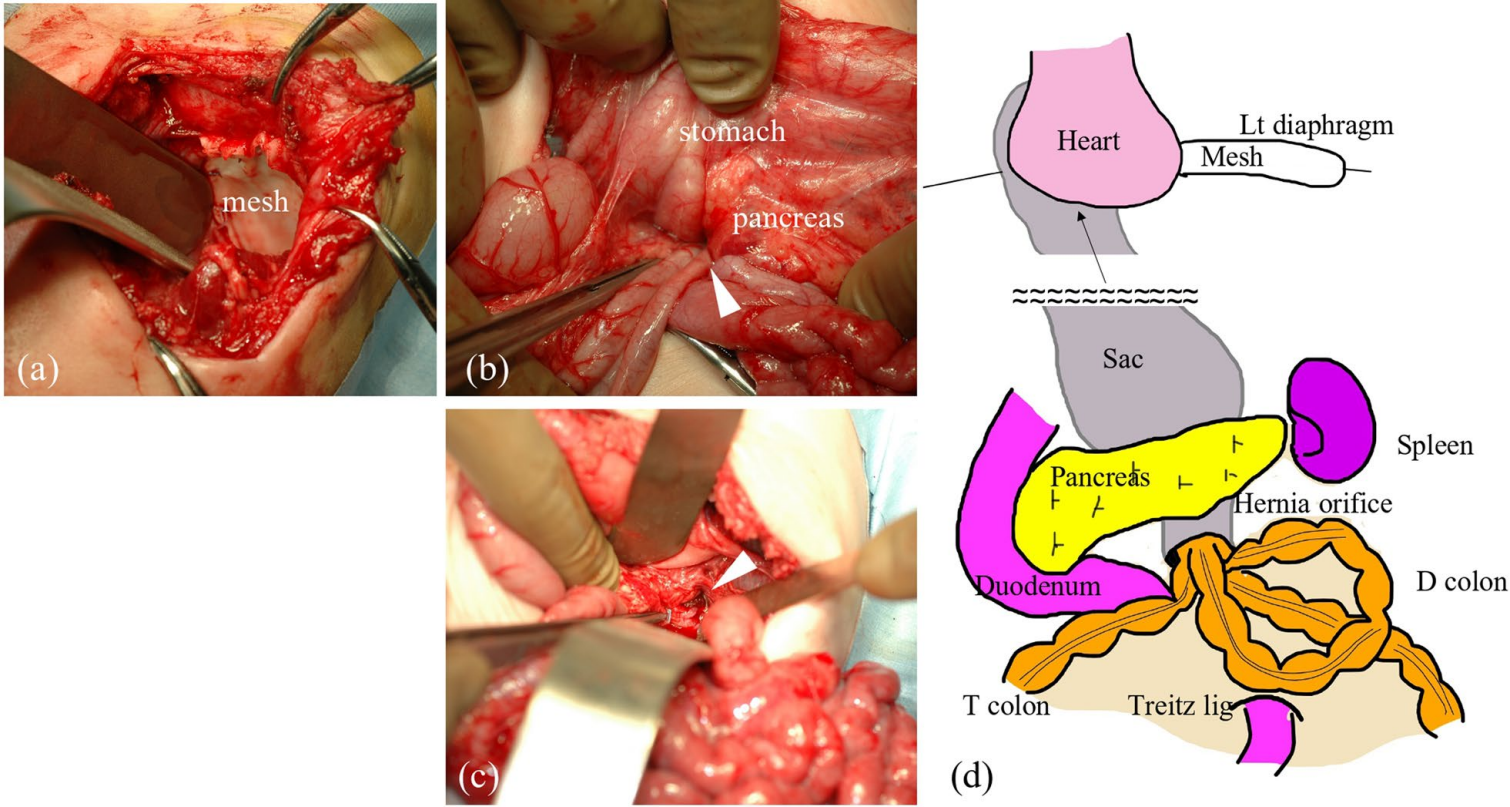
Intraoperative findings in case 1. **a** No apparent defect hole at the diaphragm. **b** Transverse colon protrudes into the absence of retropancreatic fascia (arrowhead). **c** The absent site of retropancreatic fascia is suture closed. **d** Schema shows the hernia sac protrudes from the absence of retropancreatic fascia into the posterior mediastinum

A Japanese girl was born at a gestational age of 37 weeks, weighing 2550 g. Prenatal diagnosis of a left diaphragmatic hernia was suspected. Chest radiography revealed a left diaphragmatic hernia (Fig. 1), and laparotomic left diaphragmatic hernia repair using an artificial mesh was performed at the age of one day. The defect hole was 5.5 cm in diameter, with prolapse of the liver, stomach, spleen, intestine, and colon. The postoperative course was good; however, chest radiography at the age of 35 days showed bowel gas in the mediastinum (Fig. 2). Computed tomography revealed intestinal prolapses from the medial side of the mesh into the thoracic cavity (Fig. 3). Diaphragmatic hernia recurrence was suspected, and reoperation was performed at the age of 77 days. Operative findings showed no apparent defect holes in the diaphragm (Fig. 4a). The defect hole was finally found in the absence of the retropancreatic fascia (Fig. 4b) and was connected to the posterior mediastinum from the supramesocolic space. The prolapsed organs included the transverse and descending colons. The route of the hernias started from the retropancreatic fascia defect, went through the dorsal side of the pancreatic body, and then went into the posterior mediastinum space through the aortic hiatus. The aorta was moved to the ventral side of the herniated sac by the herniated sac. As a result, the route of the hernias was present just in front of the vertebral body, behind the aorta, and at the medial side of the artificial mesh. There was no apparent defect hole in the diaphragm with the artificial mesh. We cut the parietal pleura on the mediastinal space that was on the medial side of the artificial mesh. The aorta was present on the ventral side of the mediastinal space. The mediastinal space was closed by suturing the spinal periosteum and artificial mesh with a 3-0 non-absorbable suture. The defect hole in the pancreatic body was sutured (Fig. 4c). A schema of this hernia is shown in Fig. 4d. The postoperative course was uneventful.

### Case 2

**Fig. 5:**
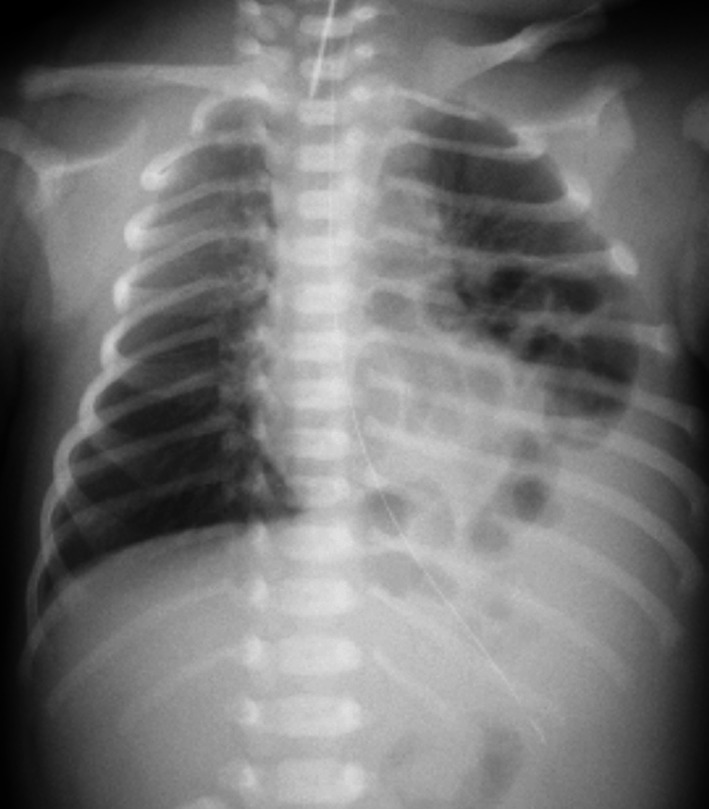
Chest radiograph in case 2. Chest radiograph shows left diaphragmatic hernia

**Fig. 6:**
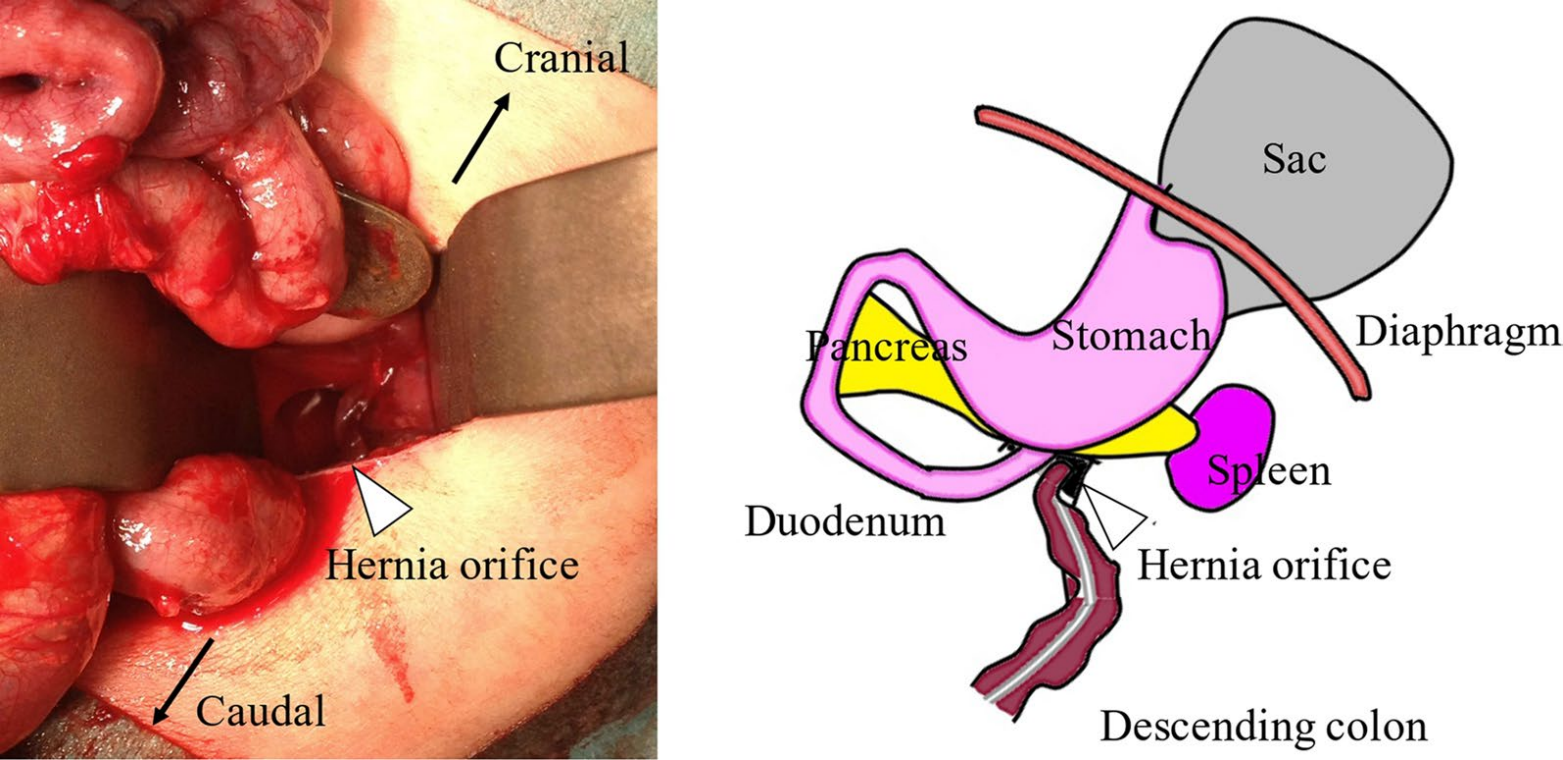
Intraoperative findings and its schema. Schema shows that the herniated orifice is at the caudal site of the pancreatic body. Hernia sac protrudes from the orifice into the extra-plural space

A Japanese boy was born at a gestational age of 40 weeks, weighing 3502 g, with a prenatal diagnosis of a left diaphragmatic hernia. Chest radiography revealed a left diaphragmatic hernia at the age of one day (Fig. 5), and emergency laparotomy was performed at the age of two days. Operative findings showed no defect hole in the diaphragm, and no intestine was observed in the abdominal cavity. After close observation of the abdominal cavity, the intestine was found around the pancreatic body and manual reduction of the intestine was performed. The defect hole existed in the absence of the retropancreatic fascia and was connected to the extra-pleural space as a result (Fig. 6). The prolapsed organs include the jejunum through the descending colon. The route of the hernia was similar to that in case 1. The defect hole in the pancreatic body was sutured and closed with a non-absorbable thread. Malrotation was incidentally detected; hence, additional band resection and prophylactic appendectomy were also performed. The postoperative course was uneventful.

## Discussion

Internal hernia is characterized by protrusion of the abdominal viscera through a peritoneal or mesenteric hernia orifice into a compartment in the abdominal cavity. There are various types of internal hernias; however, the type of internal hernia with herniated organs in the retroperitoneal space is called a retroperitoneal hernia.

**Fig. 7:**
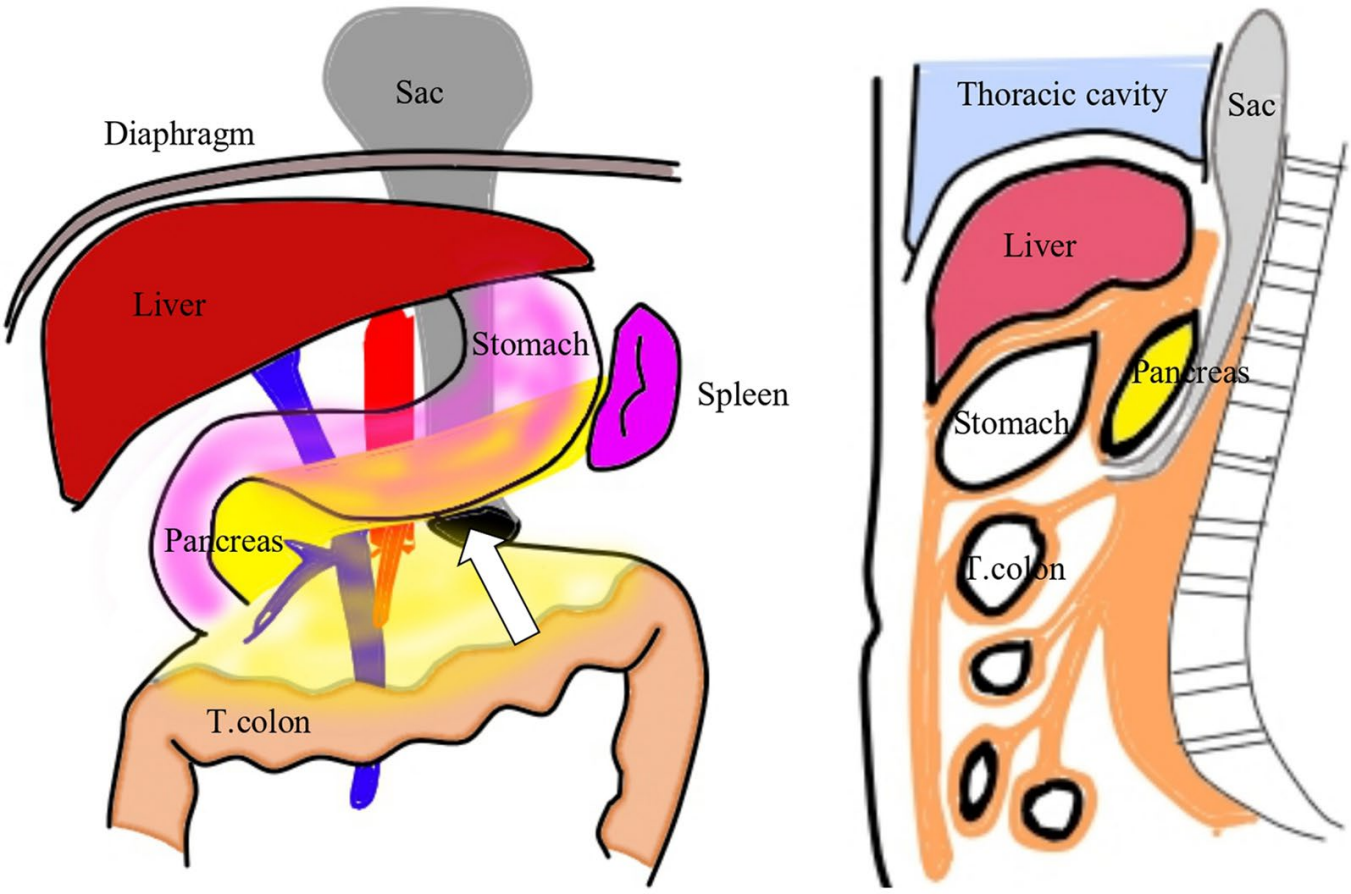
Retropancreatic fascia hernia. Schema shows retropancreatic fascia hernia; prolapsed organs extrude into the extra-pleural space through the absent site of retropancreatic fascia around the superior mesenteric artery

We encountered two cases of retroperitoneal hernia in which the hernia orifices lacked retropancreatic fascia in the supramesocolic space, and the herniated organs in each case protruded into the mediastinum and extra-pleural space, respectively. To the best of our knowledge, there have been no reports of this type of hernia. In embryology, the ventral and dorsal pancreas occur in the fifth week of the embryo and gradually merge with the posterior peritoneum. The greater omentum and transverse mesocolon completely fuse with each other after physiologic herniation at gestational age 8–9 weeks [2]. Thereafter, Toldt fusion fascia is formed by merging the dorsal peritoneum of the pancreas with the posterior peritoneum [3]. The Toldt fusion fascia behind the pancreas is known as the retropancreatic fascia. The retropancreatic fascia is absent along the pancreas facing the superior mesenteric artery in elder individuals [4]. Considering the location of the hernia orifices in our cases, the absent site of the retropancreatic fascia was assumed to be the orifice of the internal hernia. The mechanism of retropancreatic fascia hernia formation is not known; however, it can be assumed the mechanism is similar to that of paraduodenal hernia formation. Factors involved in the formation of a left duodenal hernia are the presence of a fossa, the presence of the neck of the sac (of the inferior mesenteric vein in left duodenal hernia), and sufficient mobility of the small bowel to allow it to pass into the sac derived from this fossa [5]. In addition, an increased intra-abdominal pressure is required to initiate the hernia. In retropancreatic fascia hernia, the presence of a fossa is the absent site of the retropancreatic fascia and the presence of the neck of the sac is the pancreatic body. The herniated sac prolapses into the posterior mediastinum by intra-abdominal pressure (Fig. 7). In this scenario, there is no need for other defects such as transverse fascia and diaphragm. Since various defect sites rarely occur simultaneously, this mechanism is conceivable.

Retroperitoneal hernia includes paraduodenal, pericecal, intersigmoid, foramen of Winslow, and iatrogenic posterior peritoneal defects. Paraduodenal hernias are the most common type [6] and retroperitoneal hernias caused by posterior peritoneal defects are relatively rare. Initially, we suspected paraduodenal hernia in our cases. However, Case 2 was with malrotation and without Treitz ligament; hence, the paraduodenal site did not anatomically exist. The hernia orifices of Case 1 were apparently above the mesentery roots of the transverse colon, such as in the supramesocolic space, as the operative imaging shows; therefore, the paraduodenal hernia was impossible. Considering the aspect of congenital mechanisms, we believed that a retropancreatic fascial hernia was the cause. Our two cases were considered to have congenital mechanisms. Case 2 was prenatally diagnosed with congenital diaphragmatic hernia; hence, congenital factors were assumed to contribute to the occurrence of retropancreatic fascial hernia. Case 1 was born with a Bochdalek hernia, requiring repair of the diaphragmatic hernia. Retropancreatic fascia hernia occurred approximately 1 month after the repair. Although both congenital and acquired factors were possibly associated with hernia occurrence, congenital factors were assumed to contribute to the occurrence of retropancreatic fascia hernia because the diaphragm was treated only without intraoperative damage to the retroperitoneal space during the primary surgery for Bochdalek hernia. Bochdalek hernia in the prenatal period may conceal the symptoms of a retropancreatic fascial hernia. Repairing the Bochdalek hernia reduced the prolapsed organs, possibly resulting in the protrusion of organs from the absence of the retropancreatic fascia into the peritoneal space and the mediastinum.

We retrospectively reviewed our two patients; however, preoperative diagnosis of a retropancreatic fascial hernia was difficult. Preoperative diagnosis of a retropancreatic fascia hernia is possible with chest and abdominal CT scan; however, we do not routinely perform CT scans in almost all cases with suspected congenital diaphragmatic hernia because moving the patients to a CT room alone poses a risk to these patients. In addition, the range of a CT scan is limited to the chest region. There are three types of congenital diaphragmatic hernia: a posterolateral defect, Bochdalek-type; an anterior defect, Morgagni-type; and a central defect, septum transversum-type [7]. A posteromedial diaphragmatic hernia is very rare; hence, if preoperative imaging shows a medial diaphragmatic hernia, it can suggest the possibility of a retropancreatic fascial hernia. When one cannot detect any defect holes in the preoperative diagnosis of congenital diaphragmatic hernia, hernia orifices in the abdominal cavity need to be searched for, especially in thoracoscopy because this hernia does not extrude into the thoracic cavity but into the extra-pleural space. The treatment of retropancreatic fascial hernia involves reducing the herniated organs and closing the orifices.

## Conclusions

We report two cases of retropancreatic fascial hernia. This is a new type of congenital retroperitoneal hernia in the supramesocolic space. It may be necessary to explore for herniated orifices in the abdominal cavity when there are no defect holes in the diaphragm in cases of a preoperative diagnosis of diaphragmatic hernia.

## Data Availability

Not applicable.
